# Effect of Exercise on Diabetes-Induced Oxidative Stress in the Rat Hippocampus

**Published:** 2012-04-01

**Authors:** M Alipour, I Salehi, F Ghadiri Soufi

**Affiliations:** 1 Department of Physiology, Faculty of Medicine, Zanjan University of Medical Sciences, Zanjan, Iran; 2 Department of Physiology, Faculty of Medicine, Hamadan University of Medical Sciences, Hamadan, Iran; 3 Neurosciences Research Center, Tabriz University of Medical Sciences, Tabriz, Iran

**Keywords:** Oxidation Stress, Diabetes, Treadmill Exercise, Apoptosis, Hippocampus

## Abstract

**Background:**

Oxidative stress forms the foundation for the induction of multiple cellular pathways which can lead to the complications of diabetes mellitus that the most debilitating ones are diseases of the nervous system. In this study, we evaluated whether treadmill running could alleviate oxidative stress and apoptosis rate in the hippocampus of streptozotocin- induced diabetic rats.

**Methods:**

Forty male Wistar rats were randomly divided into four groups (n=10): Control group (CR), exercised group (CE), diabetic group (DR) and diabetic-exercised group (DE). Diabetes was induced by injection of streptozotocin in male rats. All rats in the trained group run on a rodent motor-driven treadmill for eight weeks. At the end of eight weeks, hippocampi of animals were immediately removed on ice and kept frozen. The light supernatant was taken and stored at -80°C. They were used for determination of antioxidant enzymes and TBARs level. Index of apoptosis was detected by cell death detection ELISA Kit.

**Results:**

Levels of TBARs in DR and DE groups were significantly higher than CR group. SOD and GPx activities significantly increased in CE group and decreased in DR group. CAT activity significantly decreased in DR group versus CR group. The apoptosis rate significantly increased and decreased in DR and CE groups respectively compared to CR.

**Conclusion:**

Exercise had beneficial effects in the diabetic exercised rats, possibly in part because of alterations in the ability to adapt to exercise-induced oxidative stress.

## Introduction

Diabetes mellitus (DM) is a significant healthcare concern worldwide that affects more than 165 million individuals leading to cardiovascular disease, nephropathy, retinopathy, and widespread disease of both the peripheral and central nervous systems. Disease of the nervous system can become one of the most debilitating complications and affect sensitive cognitive regions of the brain, such as the hippocampus that modulates memory function, resulting in significant functional impairment and dementia. Oxidative stress forms the foundation for the induction of multiple cellular pathways that can ultimately lead to both the onset and subsequent complications of DM.[1]

Defects in hippocampal synaptic plasticity and transmission resulting in impairment of learning and memory is one the central nervous system complications of diabetes mellitus.[2] Increasing evidence in both experimental and clinical studies suggests that oxidative stress plays a central role in the onset and subsequent complications of diabetes mellitus like neuropathy.[3]

Reactive oxygen and nitrogen species, namely superoxide, hydroxyl free radicals, hydrogen peroxide and nitric oxide (NO) are produced as a consequence of the physiological metabolic reactions in the central nervous system. Superoxide radicals are converted to hydrogen peroxide by superoxide dismutase (SOD) and hydrogen peroxide is converted to water by glutathione peroxidase (GPX) and/or catalase.[4] Hydrogen peroxide can cross all membranes and lead to hydroxyl radical formation at more distant sites. The hydroxyl radical can cause lipid peroxidation, which damages cellular organelles and membranes, leading to cell death.[4] NO is rapidly broken down by superoxide radicals, and its activity is increased in the presence of SOD.[5]

The brain is particularly vulnerable to oxidative injury because of its high rate of oxygen consumption, intense production of reactive radicals, and high levels of transition metals, such as iron, that catalyze the production of reactive radicals. Moreover, neuronal membranes are rich in poly unsaturated fatty acids, which are a source of lipid peroxidation.[6] Free radicals are formed disproportionately in diabetes by glucose oxidation, non-enzymatic glycation of proteins, and the subsequent oxidative degradation of glycated proteins. Abnormally high levels of free radicals and the simultaneous decline of antioxidant defense mechanisms can lead to damage of cellular organelles and enzymes, increased lipid peroxidation, and development of insulin resistance. These consequences of oxidative stress can promote the development of complications of diabetes mellitus.[7]

Some studies suggest that regular moderate physical exercise has beneficial effects on the brain.[8] Exercise can stimulate neurogenesis in the hippocampus and improve learning and memory.[9][10][11] It has reported that exercise can enhance cell proliferation in the hippocampus of intact and diabetic animals.[12][13] Recently, our colleagues in our laboratory depicted that exercise training may prevent synaptic plasticity impairment in hippocampus of streptozotocin- induced diabetic rats.[2] It is believed that regular exercise enhances antioxidant enzymes activities, increases resistance against oxidative stress and therefore reduces cellular oxidative damage.[14]

According to our knowledge, there is little evidence about the effect of regular exercise in the hippocampus of diabetic rats with oxidative stress and apoptosis approach. In the present study, we examined whether treadmill running could alleviate oxidative stress and apoptosis rate in the hippocampus of streptozotocin (STZ)-induced diabetic rats.

## Materials and Methods

Forty male Wistar rats (two mounts old and body mass index of 190±12 g) were obtained from laboratory animal house of Tabriz University of Medical Sciences. Animals were housed at room temperature (23±2°C) with 12:12-h light/dark cycles and had free access to food and water. The study protocol was designed in accordance with the guide for the care and use of laboratory animals published by the US National Institutes of Health (NIH Publication, revised 1996) and approved by the Ethics Committee for the Use of Animals in Research of the Tabriz University of Medical Sciences.

Rats were randomly divided into four groups (n=10 for each group); the control-rest group (CR), the control-exercised group (CE), the diabetic-rest group (DR) and the diabetic-exercised group (DE). All rats in the trained group run on a rodent motor-driven treadmill (Danesh Yakhteh Co., Tabriz, Iran) for eight weeks and performed 17 m/min with a 0% slope, 40 min/day and 7 days/week.[2] While trained rats were undergoing the training programs, sedentary control rats were placed on the non-movement treadmill for 10 minutes daily to familiarize themselves with treadmill and handling. For induction of diabetes, 50 mg/kg of streptozotocin (STZ; Sigma, St.Louis, MO, USA) was injected intraperitoneally and in the control animals were injected with saline. Blood glucose levels were determined 2 days after STZ injection using a blood glucose tester (Arkray, Kyoto, Japan). Animals with blood glucose level of 300 mg/dl or higher were used as the diabetic (Table 1). All the diabetic rats were killed eight weeks after induction of diabetes.

**Table 1:** Mean blood glucose concentrations (mg/dl) in different days after STZ-treatment, (The values represent mean±SD of 10 animals per group).

	**2 days**	**7 days**	**15 days**	**28 days**
CR	106.86±04.66	111.81±03.73	107.03±04.33	110.23±05.19
CE	108.27±04.16	105.38±05.56	109.88±04.41	109.76±04.36
DR	402.33±32.42^a^	395.97±29.51^a^	400.35±27.02^a^	372.41±34.03^a^
DE	386.29±27.11^a^	390.04±26.12^a^	382.25±18.70^a^	388.18±30.54^a^

^a^ p<0.01 versus control rest group (CR), CE: The control-exercised group, DE: The diabetic-rest group, DE: The diabetic-exercised group.

Twenty-four hours after the last exercise-training session of CE and DE groups, all rats were anaesthetized with ether, and killed by decapitation. The hippocampus was quickly removed and was homogenized on ice in 1 ml of ice-cold lysis buffer (10 mM NaCl, 1.5 mM MgCl2, 20 mM HEPES, 20% glycerol, 0.1% Triton X-100, 1 mM dithiothreitol, pH=7.4). The homogenates were centrifuged at 1000 rpm for 1 min at 4°C. The supernatants contained the cytoplasmic protein fraction which were collected and protease inhibitor cocktail (104 mM AEBSF, 0.08 mM aprotinin, 2 mM leupeptin, 4 mM bestatin, 1.5 mM pepstatin A, and 1.4 mM E-64) (P8340, Sigma-Aldrich, St Louis, MO, USA) was added to them and stored at -80ºC until use. Protein concentration of the supernatant was estimated using the Bradford technique.[15]

Lipid peroxidation was analyzed by measuring thiobarbituric acid-reactive substances (TBARs) in homogenates, as previously described by Draper and Hadley.[16] Briefly; the samples were mixed with 1 mL 10% trichloroacetic acid and 1 mL 0.67% thiobarbituric acid. Then the samples were heated in a boiling water bath for 15 min and butanol (2: 1, v: v) was added to the solution. After centrifugation (800g, 5 min), TBARs were determined from the absorbance at 535 nm.

The glutathione content of the hippocampus was determined by the method of Griffith in homogenates prepared as follows. The hippocampus was homogenized in 5 vol. of 1% trichloroacetic acid (w/v), after which the homogenates were centrifuged at 18000 g for 10 min. For total glutathione determination, an aliquot of the supernatant was diluted 1/50 and 100 µL of this preparation was added to a mixture containing 0.21 mM NADPH, 0.6 mM 5,5¢-dithiobis (2-nitrobenzoic acid), 5 mM EDTA, and 0.5 U of glutathione reductase in 100 mM sodium phosphate buffer, pH=7.5, in a final volume of 1 mL. The absorbance at 412 nm was recorded. Total GSH content was calculated by comparison of the rate observed with a standard curve generated with known amounts of glutathione.[17]

Superoxide dismutase (SOD) activity was determined using a RANSOD kit (Randox labs. Crumlin, UK) as described by Delmas-Beauvieux et al.[18] SOD activity was measured at 505 nm on a spectrophotometer on supernatant. In this method, xanthin and xanthin oxidase were used to generate superoxide radicals that reacted with 2-(4-iodophenyl)-3-(4-nitrophenol)-5-phenyl tetrazolium chloride (ITN) to form a red formazan dye. Concentrations of substrates were 0.05 mmol/L for xanthin and 0.025 mmol/L for INT. SOD was measured by the degree of inhibition of this reaction.

Glutathione peroxidase (GPx) activity was determined using a RANSEL kit (Randox labs.), according to the method of Paglia and Valentine.[19] GPx catalyses the oxidation of glutathione (at a concentration of 4 mmol/L) by cumene hydroperoxide. In the presence of glutathione reductase (at a concentration 0.5 units/L) and 0.28 mmol/L of NADPH, the oxidized glutathione was immediately converted to the reduced form with a concomitant oxidation of NADPH to NAD+. The decrease in absorbance at 340 nm was measured using a spectrophotometer.

Catalase activity (CAT) was measured as previously described by Aebi.[20] The decomposition of H2O2 was followed directly by the decrease in absorbance at 240 nm and 20oC. Previously, hippocampus homogenate aliquots were centrifuged at 1000 g and 4oC for 10 min. The adequate amount of supernatants (60 µL equivalent to 1.5 mg tissue wet weight) was added to a reaction mixture that contained 0.002% Triton X-100, 0.1 mm EDTA, 0.5 m potassium phosphate buffer, pH=7.0, and 15 mm H2O2 in 1 mL final volume. Activity was calculated with the initial 30s decomposition rate.

Cell death detection ELISA kit (1544675, Roche Molecular Biochemicals, Mannheim, Germany) was used to quantitatively detect the cytosolic histone-associated DNA fragmentation, according to manufacturer’s instructions. Briefly, the extracted cytoplasmic fractions of hippocampus were used as an antigen source in a sandwich ELISA with a primary antihistone mouse monoclonal antibody coated to the microtiter plate and a second anti-DNA mouse monoclonal antibody coupled to peroxidase. The amount of peroxidase retained in the immunocomplex was determined photometrically by incubating with 2,2'-azino-di-[3- ethylbenzthiazoline sulfonate] (ABTS) as a substrate for 10 min at 20°C. The change in color was measured at a wavelength of 405 nm. All measurements were performed in duplicate, with control and trained samples analyzed on the same microtiter plate in the same setting. The OD405 reading was then normalized to the total amount of protein in the sample. The data were reported as an apoptotic index (OD405/mg protein) to indicate the level of cytosolic mono and oligonucleosomes.

After testing for normality of the distribution by Shapiro-Wilk test, One-way ANOVA (SPSS software, Version 16.0, Chicago, IL, USA) was used for analysis of between group differences in variables including TBARs, antioxidant enzymes and apoptotic index. When a significant p-value was obtained, the Tukey post-hoc test was employed to determine the differences between groups. A level of p<0.05 was selected to indicate statistical significance. Data were expressed as mean±SD.

## Results

**Fig. 1:**
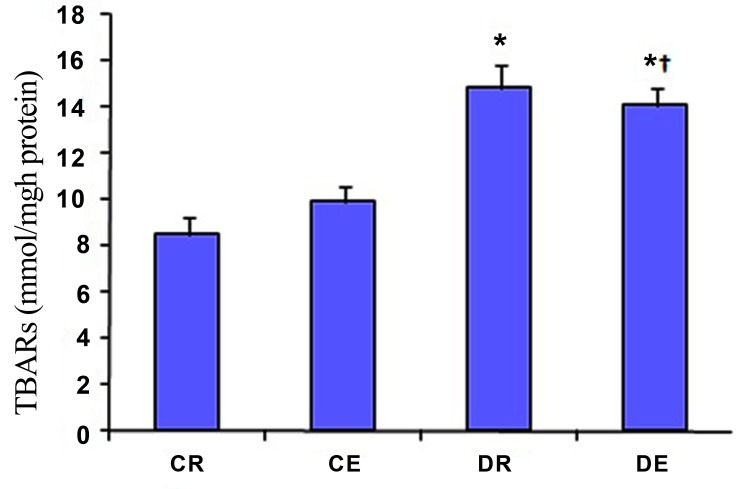
Effects of diabetes mellitus and exercise on TBARs levels in the rat hippocampus. The values represent mean±SD of 10 animals per group. *p<0.01 versus control group (CR). †p<0.01 versus exercised group (CE). TBARs: Thiobarbituric acid-reactive substances.

Figure 1 depicts that the levels of TBARs in DR and DE groups were higher (p=0.001 for both) than CR group (the effect of diabetes) but the levels of TBARs showed no change in CE group compared to CR group. Although the hippocampus GSH levels did not change in CE and DE groups versus CR group (Table 2), but its levels reduced in DR group versus (p=0.009) versus CR and DE groups (p=0.004).

**Table 2:** Effects of diabetes mellitus and exercise on activity of antioxidant enzymes and levels of GSH in the rat hippocampus (The values represent mean±SD of 10 animals per group).

**Groups**	**SOD _(U/mg protein)_**	**GPX_(U/mg protein)_**	**CAT_(mmol/mg protein)_**	**GSH _(mg/mg protein)_**
CR	9.36±0.38	3.10±0.23	30.24±0.66	0.29±0.01
CE	14.45±1.38^a^	4.63±0.52^a^,^b^	35.57±0.52^a^	0.28±0.02
DR	5.35±0.66^b^,^d^	2.06±0.16^b^,^d^	22.49±1.17^a^,^d^	0.24±0.01^a^,^d^
DE	7.76±0.41^b^,^c^^d^	3.06±0.24^c^^d^	28.53±2.18^a^,^d^	0.28±0.02^a^

^a^ p<0.01 and

^b^ p<0.05 versus control group (CR).

^c^ p<0.05 versus diabetic rest group (DR).

^d^ p<0.01 versus exercised group (CE). SOD: Superoxide dismutase, GPX: glutathione peroxidase, CAT: Catalase and GSH: glutathione.

Our results showed that the hippocampus SOD activity increased in CE group (p=0.001) and decreased in DR group (p=0.004) compared to CR group (Table 2). Although SOD activity of DE group was lower than CR group (p=0.032), but it was higher than DR group (p=0.041).

Like the SOD activity, the hippocampus GPX activity increased in CE group (p=0.009) and decreased in DR group (p=0.019). Although GPX activity did not change in DE group versus CR group, its activity was higher than DR group (p=0.029).

CAT activity decreased in DR group versus CR group in the hippocampus of the rats (p=0.001). However, this enzyme activity increased in CE group versus CR group (p=0.004). CAT activity did not change in DE group compared to CR group but it was significantly higher than DR group (p=0.002).

**Fig. 2:**
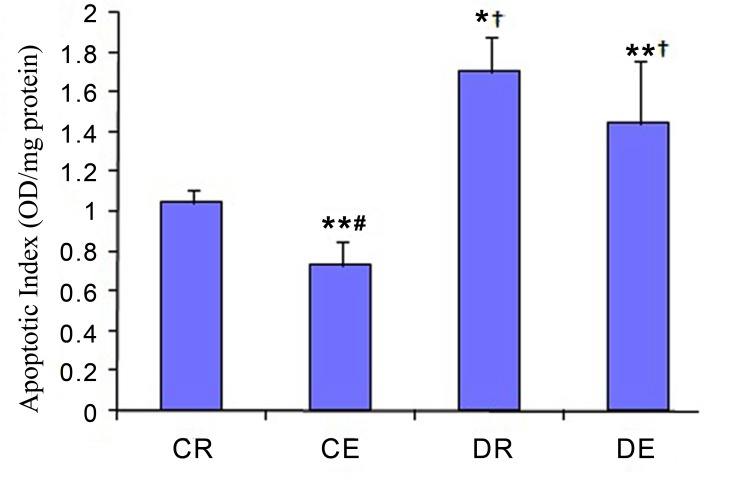
Effects of diabetes mellitus and exercise on apoptosis rate in the rat hippocampus. The values represent mean±SD of 10 animals per group. *p<0.01 and **p<0.05 versus control group (CR). #p<0.01 versus diabetic rest group (DR). †p<0.01 versus exercised group (CE).

Figure 2 depicts that the apoptosis rate increased in CR group compared to DR and CE groups (p=0.004 and p=0.023 respectively). Furthermore, our results showed that the apoptosis rate in DE group was lower than DR group (however it was not significant) and was higher than CE group (p=0.009). In addition, the apoptosis rate was significantly higher in DE group when compared to CR group (p=0.032).

## Discussion

In our study, diabetes was associated with increased hippocampus lipid peroxidation, which is a well-known marker of oxidative stress, and with significant decrease in the antioxidant enzymes SOD, GPX and CAT. In addition, the diabetes caused marked increase in the level of GSH and significant increase in hippocampus apoptosis rate.

The increase in oxidative stress, demonstrated by higher level of TBARs in diabetic rats, would be expected to increase the antioxidants activities. Our findings that SOD, GPX and CAT decreased, suggest that these activities were affected by diabetes. Previously, it was shown that diabetes induced oxidative stress and diminished antioxidant enzymes activities and capacity in the brain and hippocampus respectively.[3][21][22] However, Ramanathan et al. reported an increased SOD and CAT activities with no alteration in the GPX activity 72 hr after induction of diabetes.[23] On the contrary, they showed increased CAT and decreased GPX with no change in the SOD activity after one month of diabetes induction. Antioxidant enzymes activities in our study were measured two months after diabetes induction. Lappalainen et al. reported decreased glutathione reductase and increased GPX activities with no change in CAT and SOD activities two months after diabetes induction in the rat's brain.[21] But we measured these enzymes activities in the hippocampus and these activities may differ in different brain regions.[24]

Oxidative stress is a key participant, along with metabolic compromise and excitotoxicity, in apoptotic neurodegenerative process.[25] During oxidative stress, several lipid peroxidation products were formed that were involved in neuronal death and became an immediate substrate for GSH and aggregated further oxidative stress.[6] GSH is a major intracellular antioxidant molecule and constitutes an important mechanism against oxidative stress.[21] A decrease in GSH levels is linked to apoptotic neuronal death.[26] Our results are in agreement with this idea so that the level of GSH and apoptosis rate significantly decreased and increased respectively in the hippocampus of diabetic rats versus the control group. Previously Lappalainen et al. reported that diabetes induced oxidative stress in the rat brain through GSH reduction.[21] Furthermore, Piotrowski et al. depicted that caspase-3 activity, a key enzyme in apoptotic cell death, increased in the hippocampus of diabetic rats.[27] These data showed that diabetes mellitus results in oxidative stress and probably induced apoptosis in the hippocampus.

Our study showed that non-diabetic exercised animals had higher SOD, GPX and CAT activities than control group. Moreover, apoptosis rate was significantly lower in the non diabetic exercised rats versus the control rest. TBARs levels showed no difference between non diabetic exercised and control rats which indicate that regular exercise can not affect the peroxidation index. Aksu et al., have also shown that regular exercise does not cause oxidative stress in the rat hippocampus and it has a favorable effect on hippocampus by decreasing superoxide radical formation.[28] On the other hand Lappalainen et al. reported that antioxidant enzymes activities (except of glutathione reductase) did not increase with eight weeks of exercise in the brain.[21] This discrepancy might resulted from the different methods and brain regions for assays and from the exercise protocol and the time between the last session of exercise and killing of the animals.

We also studied the effects of exercise on diabetes-induced oxidative stress and apoptosis rate in the hippocampus. Our results depicted that regular exercise prevented effectively reduction of antioxidant enzymes activities and GSH levels in the rat hippocampus. Lappalainen et al. showed that two weeks regular exercise had no effect on SOD, GPX and CAT activities in the rat brain.[21] The reason of this discrepancy is not clear but it might be due to different effects of exercise on the different regions of the brain or from the different methods for assays and from the exercise protocol.

In this study, we showed that regular exercise prevented enhancement of lipid peroxidation and apoptosis rate in the diabetic rat hippocampus. In regard to enhancement of antioxidant enzymes activities, possibly reduced oxidative stress should be responsible for prevention of apoptosis increase. Previously, involving oxidative stress in the hippocampus cells apoptosis was suggested.[29] Lee et al. showed that in the rats started treadmill exercise 24 hr after intracerebral hemorrhage and induction of diabetes, the number of apoptotic cells significantly decreased in the dentate gyrus.[13] In addition, treadmill exercise markedly enhanced cell proliferation in the dentate gyrus of the hyperglycemic rats. Based on our knowledge, this is first study which compared the effectiveness of exercise training in the hippocampus of the rats after induction of diabetes.
